# Enlargements of the Liver.—IV

**Published:** 1910-01-22

**Authors:** 


### Medicine.

### ENLARGEMENTS OF THE LIVER.—IV.

### Cirrhosis of the Liver.

Considerable confusion exists as to the classification of the different forms of cirrhosis of the liver. The terms atrophic and hypertrophic, as applied to this disease, are unfortunate and misleading, for in the so-called atrophic variety the liver is more frequently enlarged than diminished in size. The feature which is common to all forms of the disease is the permeation of the organ by fibrous tissue. The following types have been described:

1. *Alcoholic.*—(*a*) Multilobular, or portal (or so-called atrophic), Laennec's cirrhosis; (*b*) unilobular, or biliary (or so-called hypertrophic).
2. *Hanot's* cirrhosis.
3. *Hæmochromatosis.*
4. *Splenomegalic* cirrhosis.
5. *Syphilitic.*—(*a*) Intralobular, or intercellular, or pericellular; (*b*) gummatous.

*Alcoholic Cirrhosis.*—It is probable that the multilobular type of cirrhotic liver has passed through the stage of being a unilobular cirrhotic liver. That which is "unilobular" at a given date may have become "multilobular" ten years later. It is rarer for symptoms to develop in the earlier stage, so that one meets with unilobular cirrhosis less often than multilobular, both clinically and pathologically. The main symptom, if any, at the time when the hepatic fibrosis is unilobular, is jaundice. The main symptom at the time when the hepatic fibrosis is multilobular is ascites. So different do the two cases appear clinically that they are usually described as entirely different types of disease instead of merely as different symptoms arising at different stages of the same disease.

The multilobular portal, or so-called atrophic cirrhosis of Laennec—the last stage of ordinary alcoholic cirrhosis—is chiefly characterised by signs of portal obstruction. Ascites, hæmatemesis, melæna, and some slight dilatation of the superficial abdominal veins. The liver may be small; it may have been observed to have been large and to have got smaller; it is much more common, however, to find the liver enlarged throughout the course of the disease. It is, therefore, obvious that the term atrophic is inapplicable. The organ is pale and very hard. The surface is nodular, the projecting portions varying in size from that of a big pin's head to that of a button mushroom. The edge is rounded, blunted, irregular, and beaded. The specific gravity of the organ is increased. On section islets of yellowish or yellowish-brown liver substance are seen projecting slightly from the surface, each protruding islet being surrounded by a depressed ring of grey fibrous tissue. The organ is usually so tough and dense that the maximum pressure that a strong man can exert with the pulp of his fingers is not sufficient to break it down, and yet the finger nail can break into it fairly easily, whilst it is so brittle that if a slice be taken and bent laterally it will tear with ease and will sometimes almost snap like a stick. On microscopical examination groups of lobules are found surrounded by dense bands of fibrous tissue, but there are at the same time finer strands of fibrous tissue to be seen surrounding each individual lobule. It thus happens that the microscopist's report is that the fibrosis is unilobular, when, macroscopically, there could be no doubt as to its being multilobular. What has happened in such a case is that the section under the microscope has not been of sufficient area to include one complete mesh of the multilobular fibrotic network, with the result that the finer unilobular fibrosis alone is seen and reported on. Macroscopically, multilobular cirrhosis is very much commoner than unilobular. Microscopically quite the reverse will seem to be the case, unless this source of fallacy is kept in mind. It is usual to find, in addition to fibrous tissue, a good deal of fatty degeneration also.

The spleen is generally enlarged to some extent; it often weighs between 10 and 15 ounces.

If the abdomen is not tightly distended with fluid the liver may be felt projecting below the costal margin; but it rarely reaches below the level of the umbilicus. The surface may be determined to be hard and nodular, especially in the epigastrium. The edge may also be found to be hard, well defined, irregular, and beaded. The superficial abdominal veins may exhibit some distension, though the caput medusæ, so often described in books, is hardly ever to be seen in practice. The tongue is furred and tremulous. In a few cases a continuous venous hum may be heard below the ensiform cartilage or over the spleen.

The chief symptoms are: Morning nausea and sickness, loss of appetite, especially for breakfast, attacks of cramp in the legs at night, constipation, epistaxis, hæmatemesis, melæna, a sallow complexion, progressive emaciation, increasing weakness, and abdominal distension from ascites without a great deal of œdema of the legs. The urine is often rich in urobilin. Jaundice is seldom present at this stage. The patient has not many months to live if the ascites has reached a stage requiring paracentesis abdominis.

The disease may exist without symptoms for a great many years. Cases have occurred in which former tipplers have been teetotal for upwards of 20 years and yet have died of the ascitic type of hepatic cirrhosis.

(*To be continued.*)

### Alkalies in Hyperacidity.

Bicarbonate of sodium in large doses, say 60 grains, to be given in two or three ounces of water about two hours after each of the principal meals, is much more effectual than the usual small doses. The dose may be repeated every hour for three hours. Under this treatment the pain after food rapidly diminishes, the pyrosis disappears, and the bowels are moved freely.—*Dr. S. Fenwick.*

